# Integrated Sensing and Communication Target Detection Framework and Waveform Design Method Based on Information Theory

**DOI:** 10.3390/s25020465

**Published:** 2025-01-15

**Authors:** Qilong Miao, Xiaofeng Shen, Chenfei Xie, Yong Gao, Lu Chen

**Affiliations:** 1 School of Information and Communication Engineering, University of Electronic Science and Technology of China, Chengdu 611731, China; 201911012021@std.uestc.edu.cn; 2 National Key Laboratory of Wireless Communications, University of Electronic Science and Technology of China, Chengdu 611731, China; chenff93625@163.com; 3 School of Computer Science and Information Engineering, Hefei University of Technology, Hefei 230601, China; gy_eee@163.com; 4 School of Aeronautics and Astronautics, University of Electronic Science and Technology of China, Chengdu 611731, China; lchen@std.uestc.edu.cn

**Keywords:** target detection, integrated sensing and communication, information theory, OTFS, waveform design

## Abstract

Target detection is a core function of integrated sensing and communication (ISAC) systems. The traditional likelihood ratio test (LRT) target detection algorithm performs inadequately under low signal-to-noise ratio (SNR) conditions, and the performance of mainstream orthogonal frequency division multiplexing (OFDM) waveforms declines sharply in high-speed scenarios. To address these issues, an information-theory-based orthogonal time frequency space (OTFS)-ISAC target detection processing framework is proposed. This framework adopts the OTFS waveform as its fundamental signal. The target detection is implemented through a relative entropy test (RET) comparing echo signals against target presence/absence hypotheses. Furthermore, to enhance the system’s target detection capability, the iterative OTFS-ISAC waveform design (I-OTFS-WD) method which maximizes the relative entropy is proposed. This method utilizes the minorization-maximization (MM) algorithm framework and semidefinite relaxation (SDR) technique to transform the non-convex optimization problem into an iterative convex optimization problem for resolution. The simulation results demonstrate that, under sufficient sample conditions, the RET algorithm achieves a 9.12-fold performance improvement over LRT in low-SNR scenarios; additionally, the optimized waveform reduces the sample requirements of the RET algorithm by 40%, further enhancing the target detection capability of the OTFS-ISAC system.

## 1. Introduction

Modern communication and sensing systems tend to integrate functionalities to share infrastructure, improve resource utilization, and reduce consumption. Against this backdrop, the integrated sensing and communication (ISAC) system, which combines communication and sensing functionalities while sharing resources such as energy, spectrum, waveforms, and antennas, has garnered significant research attention in recent years [1,2,3,4,5,6]. The research on ISAC encompasses various aspects, including physical-layer system design, networking considerations, ISAC applications, waveform design, performance trade-offs, signal processing techniques, and other related topics [4,5].

Waveform is a key component of ISAC systems [5]. ISAC systems tend to transmit a single signal to simultaneously achieve communication and sensing functionalities, thereby improving spectral efficiency. Existing studies on ISAC waveforms are mostly focused on OFDM due to its simplicity in processing. However, under high Doppler conditions, both the communication and sensing performance of OFDM deteriorate sharply, making it difficult to meet the requirements of modern ISAC systems [7]. Orthogonal time frequency space (OTFS) modulation, a recently proposed scheme, operates in the Doppler-delay (DD) domain for modulation and demodulation [8,9,10]. Unlike OFDM, OTFS maps the transmitted signal to the DD domain. During the modulation phase, the DD domain-transmitted signal is transformed into the time–frequency (TF) domain via the Heisenberg transform and then transmitted. At the receiver, the received TF signal undergoes the Wigner transform to map it back to the DD domain, thereby recovering the transmitted signal. By doing so, OTFS minimizes the impact of time and frequency dispersion caused by high mobility, leading to more reliable communications in high-mobility conditions, such as those seen in vehicular networks and high-speed train communications. The characteristics of OTFS make fast time-varying channels sparse and separable in the DD domain, offering significant advantages for ISAC signal processing. Studies have shown that OTFS outperforms OFDM in high-mobility scenarios with lower error rates [9,10]. OTFS waveforms have also been applied to sensing systems, achieving error-free Doppler frequency estimation for high-speed targets [7,11,12,13,14]. Therefore, OTFS is considered an ideal waveform for ISAC systems.

Target detection is a core sensing functionality in ISAC systems. To enhance the target detection capability of ISAC systems, waveform design techniques can be employed to optimize transmitted signals. Traditional sensing waveform optimization methods typically aim to enhance specific aspects of system performance. For instance, ref. [15,16] designed waveforms with superior autocorrelation properties to improve target detection, and ref. [17] developed local ambiguity function shaping algorithms to minimize the weighted integral sidelobe level of the ambiguity function. Although these traditional optimization methods can enhance sensing systems’ detection performance, they do not fundamentally optimize the information retrieval capability.

Information theory is a critical tool in sensing waveform design [18,19]. The use of information-theoretic approaches in sensing waveform design can be traced back to [18], where conditional mutual information between random target responses and received signals served as the objective function. Building on this foundation, ref. [19] extended information-theoretic waveform design to multiple-input multiple-output (MIMO) radar systems to address extended targets. The primary advantage of using information theory in waveform design lies in its ability to evaluate the sensing systems’ information acquisition capability, which is directly linked to improving target recognition performance. Although information theory offers advantages for sensing waveform design, existing research applying information theory to target detection is limited. Ref. [20] employed relative entropy as the objective function for radar waveform optimization and developed a relative entropy-based detection framework. Despite this, no existing studies have specifically investigated information-theoretic waveform design tailored for OTFS-ISAC systems.

Given the advantages of ISAC systems, OTFS, and information theory, this work proposes an OTFS-ISAC target detection framework. To further improve the detection perforamce of this framework, a waveform design method is proposed to optimize the transmitting OTFS signal. The main contributions of this work are as follows:

- An OTFS-ISAC target detection framework is proposed. This framework employs OTFS waveform as its fundamental waveform, and relative entropy is the adopted criterion criterion for hypothesis testing in target detection. In this framework, the relative entropy test (RET) algorithm, which uses relative entropy to measure the divergence between echo signal distributions under target-present and target-absent hypotheses, is proposed. Comparing to the traditional likelihood ratio test (LRT) target detection algorithm, RET demonstrates significant advantages under low signal-to-noise ratio (SNR) conditions. The proposed RET algorithm is suitable for application in ISAC scenarios, particularly in low-SNR environments, such as long-range target detection and the monitoring of weak targets (e.g., small unmanned aerial vehicles).
- An iterative OTFS-ISAC waveform design (I-OTFS-WD) method is proposed. I-OTFS-WD extends the work of [20] to OTFS with additional quality of service (QoS) constraints for communication. To address the non-convex optimization problem in I-OTFS-WD, the minorization-maximization (MM) algorithm framework is utilized to convert the problem into an iterative generalized quadratically constrained quadratic program (QCQP) [21]. Additionally, the semidefinite relaxation (SDR) technique is employed to solve the generalized non-convex QCQP optimization problem [22]. The waveform optimized by I-OTFS-WD further enhances the target detection capability of the OTFS-ISAC framework. The proposed I-OTFS-WD algorithm can be applied to scenarios with even lower target echo SNR, such as unmanned aerial vehicles with stealth structures or coatings. In such cases, waveform optimization is required to enhance the performance of the OTFS-ISAC system.

The structure of the paper is outlined as follows: Section 2.1 and Section 2.2 introduce the principles of OTFS-ISAC. Section 2.3 describes the RET algorithm. Section 2.4 describes the details of I-OTFS-WD. Section 3 presents simulation results and corresponding discussions. Finally, Section 4 concludes with a summary and outlines future work prospects.

## 2. Materials and Methods

### 2.1. Basic Concepts of OTFS

In the OTFS model, the TF plane is discretized into a grid as follows:

(1) ϖ=(nT,mΔf),n=0,…,N−1,m=0,…,M−1

where *T* is the time sampling interval, and Δf is the frequency sampling interval.

The modulated TF symbols $X_{tf}$ are transmitted over an OTFS frame with a duration of $\hat{T}=NT$, occupying a bandwidth of B=MΔf. The sampling frequency of the signal is $f_{s}=B$.

Similarly, the DD plane is discretized into a grid as follows:

(2) $$\vartheta =\left(\frac{k}{NT},\frac{l}{M\Delta f}\right),k=0,\ldots ,N-1,l=0,\ldots ,M-1$$

### 2.2. The Input–Output Relationship of OTFS-ISAC

**Figure 1:**
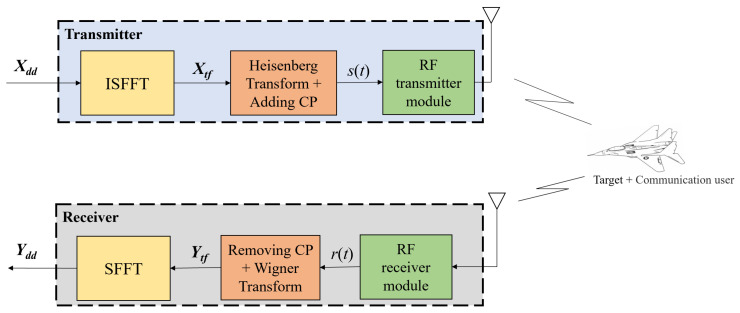
The modulation and demodulation process of the OTFS signal.

The modulation and demodulation process of the OTFS signal is illustrated in Figure 1. The transmitted symbols $X_{dd}\in C^{N\times M}$ in the DD domain are first converted into the TF symbols $X_{tf}\in C^{N\times M}$ via the inverse symplectic finite Fourier transform (ISFFT). The ISFFT transformation formula is:

(3) $$X_{tf}\left[n,m\right]=\frac{1}{\sqrt{NM}}\sum_{k^{\prime }=0}^{N-1}\sum_{l^{\prime }=0}^{M-1}X_{dd}\left[k,l\right]e^{j2\pi (\frac{kn}{N}-\frac{lm}{M})}$$

$X_{tf}\in C^{N\times M}$ is transformed into the time-domain transmitted signal s(t) through the Heisenberg transform. The Heisenberg transform formula is:

(4) $$s\left(t\right)=\frac{1}{M}\sum_{n=0}^{N-1}\sum_{m=0}^{M-1}X_{tf}\left[n,m\right]g_{tx}\left(t-nT\right)e^{j2\pi \Delta f(t-mT)}$$

where $g_{tx}$ represents the rectangular pulse shape of the transmitted signal. In OTFS-ISAC, a cyclic prefix (CP) is added to ensure the integrity of the signal symbols. The signal s(t), after undergoing RF front-end processing, is converted into an RF signal and transmitted via the antenna.

The target scattering point model in the DD domain is as follows:

(5) $$h\left(\tau ,f_{d}\right)=\sum_{p=1}^{P}\alpha_{p}\delta \left(\tau -\tau_{p}\right)\delta \left(f_{d}-f_{d_{p}}\right)$$

where *P*, $\alpha_{p}$, $\tau_{p}$, and $f_{d_{p}}$ represent the total number of target scattering points, the scattering coefficient, the delay, and the Doppler frequency *p*-th scattering point, respectively. Based on $h(\tau ,f_{d})$, the expression for the target echo signal is as follows:

(6) $$\begin{array}{cc} r(t) & =\int \int h\left(\tau ,f_{d}\right)s\left(t-\tau \right)e^{j2\pi f_{d}t}d\tau df_{d}+w\left(t\right)\\ \; & =\sum_{p=1}^{P}\alpha_{p}s\left(\tau -\tau_{p}\right)e^{j2\pi f_{d_{p}}t}+w\left(t\right) \end{array}$$

where w(t) is the additive white Gaussian noise (AWGN).

The transmitted RF signal, after being reflected by the target, returns to the receiver. After undergoing RF front-end processing, the received signal r(t) is obtained. At the receiver, the CP is removed from r(t), and the Wigner transform is applied to obtain the TF received signal $Y_{tf}\in C^{N\times M}$. The Wigner transform is given by (7) and (8):

(7) $$Y_{tf}\left(t,f\right)=\int r\left(t^{\prime }\right)g_{rx}^{*}\left(t^{\prime }-t\right)e^{-j2\pi ft^{\prime }}dt^{\prime }$$

(8) $$Y_{tf}\left[n,m\right]=Y_{tf}\left(t,f\right){|}_{t=nT,f=m\Delta f}$$

In (7), $g_{rx}\left(t\right)$ is the rectangular received pulse waveform. After applying the symplectic finite Fourier transform (SFFT) to $Y_{tf}$, the DD-domain-received symbols $Y_{dd}\in C^{N\times M}$ are obtained. The SFFT transformation is defined as follows:

(9) $$Y_{dd}\left[k,l\right]=\frac{1}{\sqrt{NM}}\sum_{k=0}^{N-1}\sum_{l=0}^{M-1}Y_{tf}\left[n,m\right]e^{-j2\pi (\frac{kn}{N}-\frac{lm}{M})}$$

After discretization and simplification, the input–output relationship of OTFS-ISAC is expressed as follows:

(10) $$Y_{dd}\left[k,l\right]=\sum_{p=1}^{P}X_{dd}\left[{\left[k-k_{p}\right]}_{N},{\left[l-l_{p}\right]}_{M}\right]\times H_{k_{p},l_{p}}\left[k,l\right]+W\left(k,l\right)$$

where $W\in C^{N\times M}$ is the AWGN matrix, $k_{p}=f_{d_{p}}N/\Delta f$, $l_{p}=\tau_{p}M/T$, ${\left[•\right]}_{N}$ denotes the modulo operation, and $H_{k_{p},l_{p}}\in C^{N\times M}$ is a factor matrix related to $k_{p}$ and $l_{p}$. The (k,l)-th element of $H_{k_{p},l_{p}}$ is calculated as:

(11) $$H_{k_{p},l_{p}}\left[k,l\right]=\frac{1}{NM^{2}}\left(H_{1}\left(k_{p},l_{p},k,l\right)+H_{2}\left(k_{p},l_{p},k,l\right)\right)e^{j2\pi f_{d_{p}}T}$$

where $H_{1}\left(k_{p},l_{p},k,l\right)$ and $H_{2}\left(k_{p},l_{p},k,l\right)$ are expressed as:

(12) $$H_{1}\left(k_{p},l_{p},k,l\right)=N\sum_{m=0}^{M-1}\sum_{m^{\prime }=0}^{M-1}e^{-j2\pi \frac{l(m^{\prime }-m)}{M}}\left\{\sum_{i^{\prime }=l_{p}}^{M-1}e^{j2\pi \frac{m^{\prime }-m+f_{d_{p}}T}{M}}\right\}$$

(13) $$H_{2}\left(k_{p},l_{p},k,l\right)=Ne^{-j2\pi \frac{k-k_{p}}{N}}\sum_{m=0}^{M-1}\sum_{m^{\prime }=0}^{M-1}e^{-j2\pi \frac{l(m^{\prime }-m)}{M}}\sum_{i^{\prime }=0}^{l_{p}}e^{j2\pi \frac{m^{\prime }-m+f_{d_{p}}T}{M}}$$

Furthermore, we perform vectorization on $H_{k_{p},l_{p}}$, $X_{dd}$, $Y_{dd}$, and W. Let $h_{k_{p},l_{p}}=\mathrm{vec}\left(H_{k_{p},l_{p}}\right)$, $x_{dd}=\mathrm{vec}\left(X_{dd}\right)$, $y_{dd}=\mathrm{vec}\left(Y_{dd}\right)$, and w=vec(W), respectively, where vec(•) represents column-wise vectorization; (9) can be expressed as:

(14) $$y_{dd}=\sum_{p=1}^{P}\alpha_{p}\Xi \left(k_{p},l_{p}\right)F\left(k_{p},l_{p}\right)x_{dd}+w$$

where $\Xi =\mathrm{diag}(h_{k_{p},l_{p}})$, and $F\left(k_{p},l_{p}\right)\in C^{N\times M}$ is the permutation matrix. For i=kM+l, the (i,j)-th element of $F(k_{p},l_{p})$ can be expressed as:

(15) $$F\left(k_{p},l_{p}\right)\left[i,j\right]=\left\{\begin{array}{c} 1,\;j={\left[k-k_{p}\right]}_{N}M+{\left[l-l_{p}\right]}_{M}\\ 0,\;otherwise \end{array}\right.$$

To simplify the notation, let $Q\left(k_{p},l_{p}\right)=\Xi \left(k_{p},l_{p}\right)F\left(k_{p},l_{p}\right)$. Thus, (14) can be rewritten as:

(16) $$y_{dd}=\sum_{p=1}^{P}\alpha_{p}Q\left(k_{p},l_{p}\right)x_{dd}+w$$

Defining $G=[Q\left(k_{1},l_{1}\right),...,Q\left(k_{P},l_{P}\right)]$, $X=I_{P}⊗x_{dd}$, and $\alpha ={\left[\alpha_{1},...,\alpha_{P}\right]}^{T}$, (16) can be expressed as:

(17) $$y_{dd}=\mathrm{GX}\alpha +w$$

### 2.3. The RET Target Detection Algorithm for OTFS-ISAC

Relative entropy can characterize the similarity between the distributions of random variables, and thus can be used to construct target detection algorithms. Since the waveform design in this paper is based on the DD domain, the subscripts “dd” for $x_{dd}$ and $y_{dd}$ will be omitted for simplicity in the following discussion.

The binary hypothesis testing model for target detection in OTFS-ISAC is as follows:

(18) $$\left\{\begin{array}{c} H_{0}:\;y=w\\ H_{1}:\;y=\mathrm{GX}\alpha +w \end{array}\right.$$

In this work, it is assumed that α is a mutually independent zero-mean complex Gaussian random vector with covariance matrix $R_{\alpha }$, and the covariance matrix of w is $R_{w}$. Clearly, $R_{H_{0}}=R_{w}$. Therefore, the probability density functions of $H_{0}$ and $H_{1}$ are given by:

(19) $$f_{H_{0}}\left(y\right)=\frac{1}{\pi^{NM}\det\left(R_{w}\right)}\exp\left(-y^{H}R_{w}^{-1}y\right)$$

(20) $$f_{H_{1}}\left(y\right)=\frac{1}{\pi^{NM}\det\left(R_{H_{1}}\right)}\exp\left(-y^{H}R_{H_{1}}^{-1}y\right)$$

where $R_{H_{1}}=\mathrm{GX}R_{\alpha }X^{H}G^{H}+R_{w}$. In this work, it is assumed that the receiver has prior knowledge of $R_{\alpha }$ and $R_{w}$. This is reasonable because $R_{\alpha }$ and $R_{w}$ can be estimated through previously received signals.

Therefore, the relative entropy between $H_{0}$ and $H_{1}$ is given by:

(21) $$\begin{array}{cc} D(H_{0}||H_{1}) & =\int f_{H_{0}}\left(y\right)\ln\frac{f_{H_{0}}\left(y\right)}{f_{H_{1}}\left(y\right)}dy\\ & =\ln\det\left(R_{H_{1}}\right)-\ln\det\left(R_{w}\right)+\mathrm{tr}\left(R_{H_{1}}^{-1}R_{w}\right)+NM \end{array}$$

In (21), the property $E_{H_{0}}\left(y^{H}R_{H_{1}}^{-1}y\right)=\mathrm{tr}\left(R_{H_{1}}^{-1}R_{H_{0}}\right)$ is used.

Therefore, by estimating the covariance matrix ${\hat{R}}_{y}$, we can calculate $D(H_{y}||H_{0})$ and $D(H_{y}||H_{1})$ through (21). If $D(H_{y}\left|\right|H_{1})>D(H_{y}\left|\right|H_{0})$, it indicates that the distribution of the received signal is more similar to $H_{0}$, meaning that there is no target in the received signal; otherwise, the target is present. The RET target detection algorithm for OTFS-ISAC is summarized in Algorithm 1.
**Algorithm 1:** The RET Target Detection Algorithm for OTFS-ISAC**Input**: y, $R_{H_{1}}$, $R_{w}$**Output**: Indicator L for the presence of the targets Estimate ${\hat{R}}_{y}$ based on y;Calculate $D(H_{y}||H_{0})$ and $D(H_{y}||H_{1})$ through (21);If $D(H_{y}\left|\right|H_{1})<D(H_{y}\left|\right|H_{0})$, L=True;Otherwise, L=False;Return L.

### 2.4. The I-OTFS-WD Algorithm

To enhance the target detection capability, it is necessary to increase the distinguishability between the distributions of $H_{0}$ and $H_{1}$. Therefore, the waveform design problem is modeled as an optimization problem that maximizes the relative entropy.

#### 2.4.1. Design of the Objective Function

We take $D(H_{0}||H_{1})$ as the objective function for the waveform design problem. Based on the OTFS-ISAC input–output relationship, (21) can be rewritten as:

(22) $$\begin{array}{cc} \; & D(H_{0}\left|\right|H_{1})=\int f_{H_{0}}\left(y\right)\ln\frac{f_{H_{0}}\left(y\right)}{f_{H_{1}}\left(y\right)}dy\\ \; & =\ln\det\left(\mathrm{GX}R_{\alpha }X^{H}G^{H}+R_{w}\right)-\ln\det\left(R_{w}\right)+\mathrm{tr}\left({\left(\mathrm{GX}R_{\alpha }X^{H}G^{H}+R_{w}\right)}^{-1}R_{w}\right)+NM \end{array}$$

Ignoring the constant terms, the objective function can be re-expressed as:

(23) $$\mathrm{obj}=\ln\det\left(\mathrm{GX}R_{\alpha }X^{H}G^{H}+R_{w}\right)-\ln\det\left(R_{w}\right)+\mathrm{tr}\left({\left(\mathrm{GX}R_{\alpha }X^{H}G^{H}+R_{w}\right)}^{-1}R_{w}\right)$$

#### 2.4.2. Design of the Constraints

In this work, the constraints are the power constraint and communication QoS constraint. Specifically, assuming that the transmission power is $P_{t}$, the power constraint is:

(24) $$X^{H}X\leq PP_{t}$$

For communication, let us assume that there are $P_{C}$ channels, with channel attenuation factors $\beta ={\left[\beta_{1},...,\beta_{P_{C}}\right]}^{T}$. Then, the received signal for the communication user is:

(25) $$y_{C}=G_{C}X\beta +w_{C}$$

where $G_{C}=\left[Q\left({\hat{k}}_{1},{\hat{l}}_{1}\right),...,Q\left({\hat{k}}_{P_{C}},{\hat{l}}_{P_{C}}\right)\right]$, and ${\hat{k}}_{i}$ and ${\hat{l}}_{i}$ represent the Doppler tap and delay tap of the *i*-th channel, respectively. $w_{C}$ is the communication AWGN, with its covariance matrix denoted as $R_{w_{C}}=\sigma_{w_{C}}^{2}I_{NM}$.

To ensure communication quality, the received signal-to-noise ratio (SNR) for the communication user must meet a minimum requirement. Therefore, the communication constraint is as follows:

(26) $$\frac{\beta^{H}X^{H}{\left(G_{C}\right)}^{H}G_{C}X\beta }{NM\sigma_{w_{C}}^{2}}\geq \eta$$

where η represents the minimum communication SNR requirement.

#### 2.4.3. Solution to the Waveform Design Optimization Problem

Based on Section 2.4.1 and Section 2.4.2, the optimization problem for the OTFS-ISAC waveform design, which maximizes relative entropy, is expressed as follows:

(27) $$P_{0}:\left\{\begin{array}{cc} \max_{X} & \;D(H_{0}||H_{1})\\ s.t. & \;X^{H}X\leq PP_{t}\\ \; & \;\frac{\beta^{H}X^{H}{\left(G_{C}\right)}^{H}G_{C}X\beta }{NM\sigma_{w_{C}}^{2}}\geq \eta \end{array}\right.$$

$P_{0}$ is a non-convex optimization problem; in this work, the MM framework is used to address it. First, the lndet(•) term in the objective function (23) is handled.

**Lemma** **1.** *The following conclusion holds for the lndet(•) term in (23):*

(28) $$\begin{array}{cc} A(U_{1}) & =\ln\det\left({\mathrm{GXR}}_{\alpha }X^{H}G^{H}+R_{w}\right)-\ln\det\left(R_{w}\right)\\ \; & =\ln\det(V_{1}U_{1}^{-1}V_{1}^{H}) \end{array}$$

*where $V_{1}=\left[I_{P},0_{P\times NM}\right]\in C^{P\times (P+NM)}$, $U_{1}\in C^{\left(P+NM)\times (P+NM\right)}$ and can be expressed as:*

(29) $$U_{1}=\left[\begin{array}{cc} I_{P\times P} & R_{\alpha }^{\frac{1}{2}}X^{H}G^{H}\\ \mathrm{GX}R_{\alpha }^{\frac{1}{2}} & R_{w}+\mathrm{GX}R_{\alpha }X^{H}G^{H} \end{array}\right]$$

*The proof of Lemma 1 is presented in Appendix A.*

According to Lemma 1 in [20], $A(U_{1})$ is a convex function with respect to $U_{1}$. According to [23], $A(U_{1})$ can be lower-bounded by its supporting hyperplane, Thus, the following conclusion can be drawn:

(30) $$\begin{array}{cc} A(U_{1}) & \geq A\left(U_{1}^{\left(t\right)}\right)+\mathrm{tr}\left(D\left(U_{1}^{\left(t\right)}\right)\left(U_{1}-U_{1}^{\left(t\right)}\right)\right)\\ \; & \geq A\left(U_{1}^{\left(t\right)}\right)+\mathrm{tr}\left(D\left(U_{1}^{\left(t\right)}\right)U_{1}\right)-\mathrm{tr}\left(D\left(U_{1}^{\left(t\right)}\right)U_{1}^{\left(t\right)}\right) \end{array}$$

where $U_{1}^{\left(t\right)}$ represents $U_{1}$ at the *t*-th iteration, and $D(U_{1}^{\left(t\right)})$ is the conjugate gradient matrix of $A(U_{1})$ at the *t*-th iteration of the MM algorithm, expressed as:

(31) $$D\left(U_{1}^{\left(t\right)}\right)=-{\left(U_{1}^{\left(t\right)}\right)}^{-1}V_{1}^{H}{\left(V_{1}{\left(U_{1}^{\left(t\right)}\right)}^{-1}V_{1}^{H}\right)}^{-1}V_{1}{\left(U_{1}^{\left(t\right)}\right)}^{-1}$$

The matrix $D(U_{1}^{\left(t\right)})$ is partitioned as follows:

(32) $$D\left(U_{1}^{\left(t\right)}\right)=\left[\begin{array}{cc} D_{11} & D_{12}\\ D_{12}^{H} & D_{22} \end{array}\right]$$

Then, in (30), $\mathrm{tr}(D\left(U_{1}^{\left(t\right)}\right)U_{1})$ can be simplified as:

(33) tr(D(U1(t))U1)=tr(2Re(D12GXRα12)+D22(Rw+GXRαXHGH)+D11)=2Re(tr(D12GXRα12)+D22(Rw+GXRαXHGH)+D11)

Combining (33), (30) can be rewritten as:

(34) A(U1)≥c1+2Re(tr(D12GXRα12))+tr(D22GXRαXHGH)

where $c_{1}$ is expressed as:

(35) $$c_{1}=\ln\det\left(V_{1}{\left(U_{1}^{\left(t\right)}\right)}^{-1}V_{1}^{H}\right)+\mathrm{tr}\left(D_{11}+D_{22}R_{w}-DU_{1}^{\left(t\right)}\right)$$

Using properties $\mathrm{tr}\left(A^{T}B\right)={\mathrm{vec}}^{T}\left(A\right)\mathrm{vec}\left(B^{T}\right)$, $\mathrm{tr}\left(\mathrm{ABCD}\right)={\mathrm{vec}}^{T}\left(D\right)\left(A⊗C\right)\mathrm{vec}\left(B\right)$, and ${\left(R_{\alpha }^{\frac{1}{2}}\right)}^{H}=R_{\alpha }^{\frac{1}{2}}$, the second term in (34) can be reformulated as:

(36) 2Re(tr(D12GXRα12))=2Re(tr(Rα12D12GX)H)=2Re(tr(XHGHD12HRα12))=2Re(x^Hs^)

where $\hat{x}=\mathrm{vec}\left(X\right)$, and $\hat{s}=\mathrm{vec}\left({\left(G^{H}D_{12}^{H}R_{\alpha }^{\frac{1}{2}}\right)}^{T}\right)$

Similarly, the third term in (34) is handled as follows:

(37) $$\begin{array}{cc} \mathrm{tr}(D_{22}GXR_{\alpha }X^{H}G^{H})\; & =\mathrm{tr}(R_{\alpha }X^{H}G^{H}D_{22}GX)\\ \; & =\mathrm{tr}(R_{\alpha }X^{H}{\hat{D}}_{22}X)\\ \; & ={\hat{x}}^{H}\left(R_{\alpha }^{*}⊗{\hat{D}}_{22}\right)\hat{x} \end{array}$$

where ${\hat{D}}_{22}=G^{H}D_{22}G$. Based on (36) and (37), (34) can be rewritten as:

(38) A(U1)≥c1+2Re(x^Hs^)+x^H(Rα*⊗D^22)x^

Next, we handle the tr(•) term in (23).

**Lemma** **2.** *The following conclusion holds for the tr(•) term in (23):*

(39) $$\mathrm{tr}\left({\left(GXR_{\alpha }X^{H}G^{H}+R_{w}\right)}^{-1}R_{w}\right)=NM-\mathrm{tr}\left(R_{w}^{-1}{\left(V_{2}U_{2}^{-1}V_{2}^{H}\right)}^{-1}\right)$$

*where $V_{2}=\left[I_{NM},0_{NM\times NM}\right]\in C^{NM\times 2NM}$, $U_{2}$ is expressed as:*

(40) $$U_{2}=\left[\begin{array}{cc} GXR_{\alpha }X^{H}G^{H} & GXR_{\alpha }X^{H}G^{H}\\ GXR_{\alpha }X^{H}G^{H} & R_{w}+GXR_{\alpha }X^{H}G^{H} \end{array}\right]$$

*The proof of Lemma 2 can be found in Appendix B.*

According to Lemma 2 in [20], (38) is a convex function with respect to $U_{2}$. Therefore, ignoring the constant term in (38), we have:

(41) $$-\mathrm{tr}\left(R_{w}^{-1}{\left(V_{2}U_{2}^{-1}V_{2}^{H}\right)}^{-1}\right)\geq -\mathrm{tr}\left(R_{w}^{-1}{\left(V_{2}{\left(U_{2}^{\left(t\right)}\right)}^{-1}V_{2}^{H}\right)}^{-1}\right)+tr\left(F\left(U_{2}^{\left(t\right)}\right)\left(U_{2}-U_{2}^{\left(t\right)}\right)\right)$$

where $F(U_{2}^{\left(t\right)})$ is the matrix of the *t*-th iteration of the MM algorithm, and its expression is:

(42) $$F\left(U_{2}^{\left(t\right)}\right)=-{\left(U_{2}^{\left(t\right)}\right)}^{-1}V_{2}^{H}{\left(V_{2}{\left(U_{2}^{\left(t\right)}\right)}^{-1}V_{2}^{H}\right)}^{-1}R_{w}^{-1}{\left(V_{2}{\left(U_{2}^{\left(t\right)}\right)}^{-1}V_{2}^{H}\right)}^{-1}V_{2}{\left(U_{2}^{\left(t\right)}\right)}^{-1}$$

The matrix $F(U_{2}^{\left(t\right)})$ is partitioned as follows:

(43) $$F\left(U_{2}^{\left(t\right)}\right)=\left[\begin{array}{cc} F_{11} & F_{12}\\ F_{21} & F_{22} \end{array}\right]$$

Then, $\mathrm{tr}(F\left(U_{2}^{\left(t\right)}\right)U_{2})$ can be further simplified as:

(44) $$\mathrm{tr}\left(F\left(U_{2}^{\left(t\right)}\right)U_{2}\right)=\mathrm{tr}\left(F_{22}R_{w}\right)+{\hat{x}}^{H}\left(R_{\alpha }^{*}⊗{\hat{F}}_{1}+R_{\alpha }^{*}⊗{\hat{F}}_{2}\right)\hat{x}$$

where ${\hat{F}}_{1}=G^{H}F_{11}G$ and ${\hat{F}}_{2}=G^{H}\left(F_{12}+F_{21}\right)G$.

Similarly, the communication constraint can be vectorized as follows:

(45) $$\frac{\beta^{H}X^{H}{\left(G_{C}\right)}^{H}G_{C}X\beta }{NM\sigma_{w_{C}}^{2}}=\frac{{\hat{x}}^{H}\left(R_{\beta }^{*}⊗{\hat{G}}_{C}\right)\hat{x}}{NM\sigma_{w_{C}}^{2}}$$

where $R_{\beta }$ is the covariance matrix of the channel, and ${\hat{G}}_{C}={G_{C}}^{H}G_{C}$.

Based on (38), (44) and (45), the optimization problem at the *t*-th iteration of Equation (27) can be expressed as:

(46) P1(t):maxx^x^HΨ^x^+2Re(x^Hs^)s.t.x^Hx^≤PPtx^HΘ^x^≥η

where $\hat{\Psi }$ is expressed as:

(47) $$\hat{\Psi }=R_{\alpha }^{*}⊗{\hat{D}}_{22}+R_{\alpha }^{*}⊗{\hat{F}}_{1}+R_{\alpha }^{*}⊗{\hat{F}}_{2}$$

$\hat{\Theta }$ is expressed as:

(48) $$\hat{\Theta }=\frac{R_{\beta }^{*}⊗{\hat{G}}_{C}}{NM\sigma_{w_{C}}^{2}}$$

Furthermore, let $A={\left[S_{1}^{T},S_{2}^{T},...,S_{P}^{T}\right]}^{T}\in C^{NMP^{2}\times NM}$, where for $S_{i}$, its (i−1)NMiNM-th rows constitute the identity matrix $I_{NM}$, while all other elements are zero. Using A, we have $\hat{x}=Ax$. Then, (46) can be rewritten as:

(49) P2(t):maxxxHΨx+2Re(xHs)s.t.xHx≤PtxHΘx≥η

where $\Psi =A^{H}\hat{\Psi }A$, $s=A^{H}\hat{s}$, and $\Theta =A^{H}\hat{\Theta }A$.

Since the communication constraint in (49) is non-convex, the optimization problem remains non-convex. Noting that (49) is a generalized QCQP problem, the SDR technique from [22] can be used to solve it. According to [22], (49) can be transformed into the following convex optimization problem:

(50) $$P_{3}^{\left(t\right)}:\left\{\begin{array}{cc} \max_{ℵ} & \;\mathrm{tr}(\Delta ℵ)\\ s.t. & \;\mathrm{tr}\left(ℵ\right)\leq P_{t}+1\\ \; & \;\mathrm{tr}(\Lambda ℵ)\geq \eta \end{array}\right.$$

where $ℵ=\overset{˘}{x}{\overset{˘}{x}}^{H}$, $\overset{˘}{x}={\left[x^{T},g\right]}^{T}$, |g|=1, and the expression of Δ is:

(51) $$\Delta =\left[\begin{array}{cc} \Psi & s\\ s^{H} & 0 \end{array}\right]$$

The expression of Λ is:

(52) $$\Lambda =\left[\begin{array}{cc} \Theta & 0\\ 0 & 0 \end{array}\right]$$

According to [22], if the optimal solution of optimization problem (50) is ${ℵ}^{★}$, then the optimal $x^{★}$ is the eigenvector corresponding to the largest eigenvalue of ${ℵ}^{★}$.

## 3. Results and Discussion

The simulation parameters of this work are shown in Table 1. Without loss of generality, we set the DD domain-transmitted symbols $X_{dd}$ as a random symbol matrix with an amplitude of 1, and $R_{\alpha }=I_{P}$. These simulation parameters align with those of typical practical millimeter-wave ISAC systems. Based on the simulation parameters, the maximum detection range of the OTFS-ISAC system is 240 m. Considering that high Doppler resolution is not required for target detection tasks and to reduce processing complexity, N=16 is selected.

Firstly, we simulate the RET target detection algorithm for OTFS-ISAC. In the simulation, we select the LRT target detection algorithm with a false alarm probability of $10^{-6}$ as a baseline for comparison.

**Figure 2:**
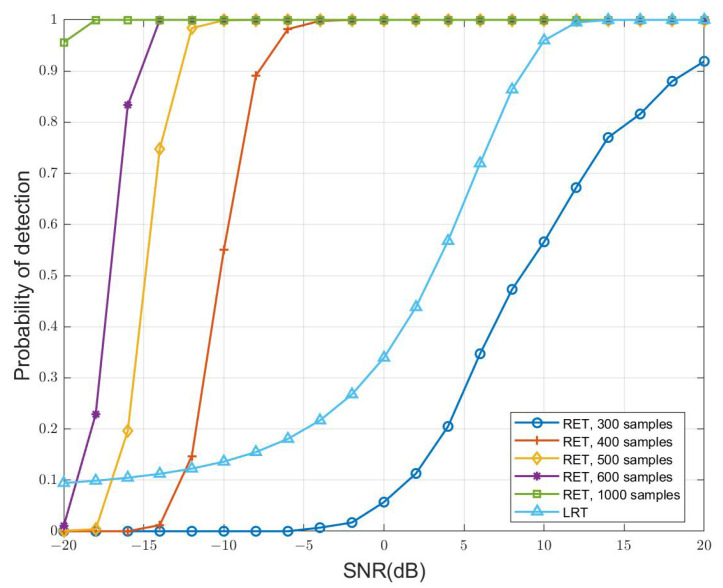
Performance curves of the RET target detection algorithm for OTFS-ISAC.

Figure 2 illustrates the variation in the detection probability of the RET target detection algorithm with respect to SNR. In the simulation, RET requires multiple echo samples to estimate ${\hat{R}}_{y}$. Therefore, we set different numbers of echo samples to observe the impact of sample size on the RET algorithm’s performance. Let the sample size be *L*. To ensure that the ${\hat{R}}_{y}$ is full-rank, it must satisfy L≥NM=256. However, when L=256, the ${\hat{R}}_{y}$ tends to be unstable and may even be singular. Therefore, we chose 300, slightly larger than 256, as the minimum simulation sample size. In traditional sensing systems based on LRT, such as radar, the detectable SNR typically ranges from 10 dB to 13 dB. However, for targets at very long distances, the two-way propagation loss is inversely proportional to the fourth power of the target distance, resulting in a significantly lower received SNR than the detectable SNR. To validate the superior target detection capability of the proposed OTFS-ISAC system, we set the minimum SNR to −20 dB.

The simulation results show that the performance of the RET algorithm depends on the number of samples used to estimate ${\hat{R}}_{y}$. When the sample size is 300, the performance of RET is inferior to LRT at any SNR level. However, as the sample size increases, the performance of RET improves significantly. With a sample size of 1000, RET achieves a detection probability of 0.956 even at an SNR of −20 dB, which is 9.12 times higher than LRT. This outcome arises because the RET algorithm heavily relies on the accuracy of ${\hat{R}}_{y}$ estimation. A larger sample size leads to a more accurate estimation of ${\hat{R}}_{y}$. Moreover, under accurate ${\hat{R}}_{y}$ estimation, the RET algorithm is less sensitive to noise. This is because the essence of RET is to “highlight” the target by evaluating the similarity between the echo signal distribution and the distributions of $H_{0}$ and $H_{1}$, rather than relying directly on SNR.

**Figure 3:**
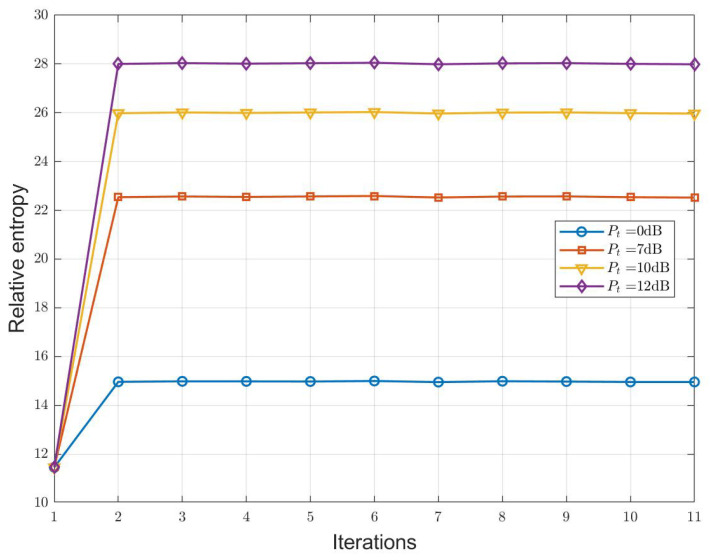
Curves of relative entropy variation during the MM iterative process of the I-OTFS-WD.

Next, we simulate the proposed I-OTFS-WD method. Figure 3 illustrates the MM iteration process of the I-OTFS-WD method under different normalized $P_{t}$ values. In this simulation, we validate the convergence speed of the I-OTFS-WD algorithm through the variation of relative entropy during the iterative process. The reason for setting different $P_{t}$ values is that practical OTFS-ISAC systems adjust the transmit power based on varying scenarios. This simulation also validates the relationship between relative entropy and $P_{t}$. It can be observed that as $P_{t}$ increases, the relative entropy achieved by the optimized waveform also increases. Additionally, Figure 3 demonstrates that the algorithm converges rapidly, requiring only a single iteration to obtain the optimal waveform.

**Figure 4:**
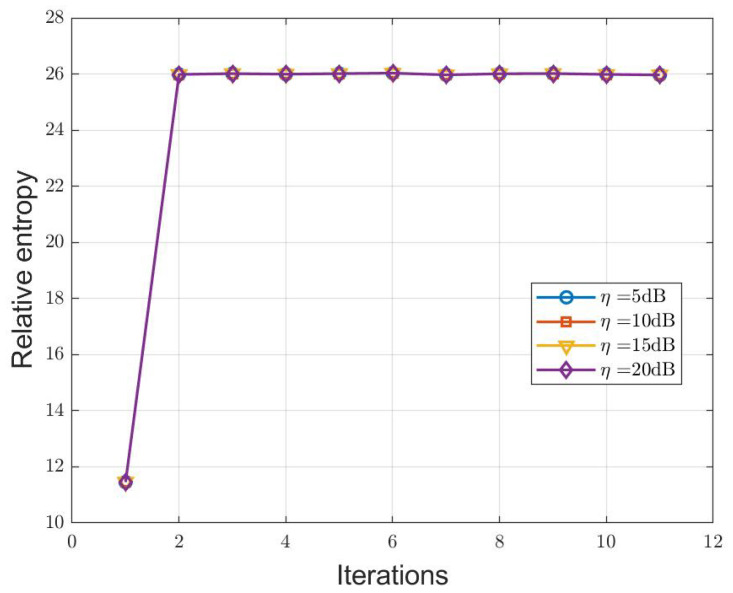
Curves of relative entropy variation during the MM iterative process of the I-OTFS-WD under different communication QoS.

Figure 4 illustrates the variation of relative entropy during the MM iteration process of the I-OTFS-WD method. In general, the sensing and communication performance of ISAC systems are mutually constrained. This simulation evaluates the impact of different communication QoS constraints on the waveforms optimized by the I-OTFS-WD algorithm. In the simulation, different communication QoS levels are set by varying η. The results in Figure 4 reveal an interesting observation: under the condition of feasible solutions, communication QoS does not affect the optimal waveform. This is because the OTFS-ISAC system proposed in this paper uses a single waveform to simultaneously accomplish both communication and target detection tasks. As long as the communication QoS is satisfied, the algorithm tends to fully utilize the transmit power, as shown in Figure 3. Under feasible conditions, the waveform that maximizes relative entropy always satisfies the communication QoS.

**Figure 5:**
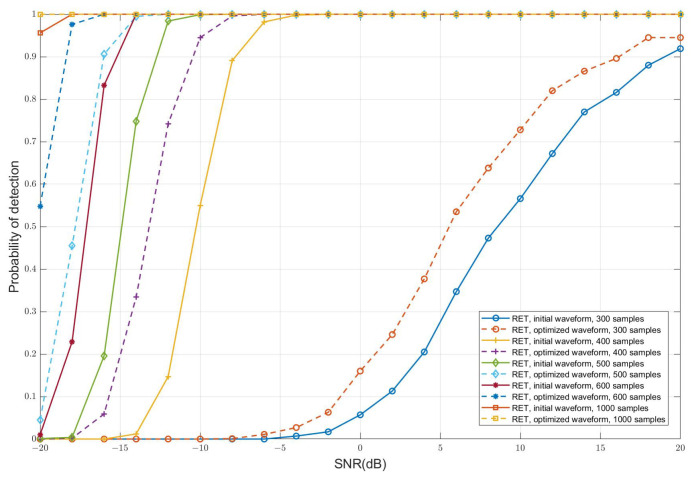
RET performance curves of the optimized waveform for OTFS-ISAC.

Figure 5 demonstrates the impact of the proposed I-OTFS-WD method on target detection performance. In this simulation, target detection is performed using the RET algorithm with both the initial waveform and the optimized waveform. This is to validate the impact of the waveforms designed by the I-OTFS-WD algorithm on detection performance and the sample size required by the RET algorithm. The simulation results show that the optimized waveform significantly enhances the system’s target detection capability for the same number of samples. For instance, at a low SNR of −18 dB, the detection probability with 600 samples using the optimized waveform reaches 0.976, which is close to the detection probability of 1.0 with 1000 samples. In other words, under comparable detection performance, the optimized waveform reduces the sample size required by the RET algorithm by 40%. The fundamental reason why the I-OTFS-WD algorithm enhances targets detection performance is as follows: The objective function of the I-OTFS-WD algorithm is to maximize the relative entropy between $H_{0}$ and $H_{1}$. By optimizing the transmitted signal vector x, the I-OTFS-WD ensures that the similarity between $H_{0}$ and $H_{1}$ is minimized in terms of both energy and direction, thereby improving the performance of the RET algorithm.

## 4. Conclusions

To enhance the detection capability of ISAC systems under low-SNR conditions and improve robustness in high-speed scenarios, this work proposes an information-theoretic OTFS-ISAC target detection framework. The framework employs OTFS waveforms to construct the RET target detection algorithm. To further improve the target detection performance of OTFS-ISAC, the I-OTFS-WD method based on maximizing relative entropy is proposed.

The simulation results indicate that the OTFS-ISAC RET target detection algorithm achieves a 9.12-fold improvement in detection probability under low-SNR conditions compared to the traditional LRT detection algorithm when sufficient samples are available. The results also show that the proposed I-OTFS-WD method converges within a single iteration and reduces the required sample size for the RET algorithm under comparable detection performance. Specifically, at low SNR, the required sample size is reduced by 40% compared to that of non-optimized waveforms. Additionally, under feasible conditions, the greater the power constraint, the better the performance of the optimal waveform, which remains unaffected by communication QoS.

The proposed OTFS-ISAC system demonstrates significant application potential in scenarios such as pedestrian monitoring in intelligent transportation systems and vehicle tracking in the Internet of Things. These applications demand the multifunctional integration of sensing and communication to reduce system costs while ensuring performance. However, limitations remain in the current system. Specifically, the RET target detection algorithm proposed in this work requires more than 400 samples to achieve satisfactory performance under low-SNR conditions, which poses practical challenges.

In future work, we will focus on improving the target detection performance of the OTFS-ISAC system under small sample conditions. Additionally, we will explore waveform design tailored to target parameter estimation in the OTFS-ISAC system.

## Figures and Tables

**Table 1:** The simulation parameters.

Symbol	Parameter	Value
$f_{c}$	Carrier frequency	77 GHz
*N*	Number of Doppler samples	16
*M*	Number of delay samples	16
*B*	Bandwidth of the signal	10 MHz
Δf	Subcarrier spacing	625 kHz
*P*	Number of targets	5

## Data Availability

The original contributions presented in the study are included in the article material; further inquiries can be directed to the corresponding author.

## Abbreviations

The following abbreviations are used in this manuscript:

ISACS	Integrated sensing and communication
OFDM	Orthogonal frequency division multiplexing
OTFS	Orthogonal time frequency space
DD	Doppler-delay
TF	Time–frequency
MIMO	Multiple-input multiple-output
RET	Relative entropy test
LRT	Likelihood ratio test
SNR	Signal-to-noise ratio
I-OTFS-WD	Iterative OTFS-ISAC waveform design
QoS	Quality of service
MM	Minorization-maximization
QCQP	Quadratically constrained quadratic program
SDR	Semidefinite relaxation
CP	Cyclic prefix
SFFT	Symplectic finite Fourier transform
ISFFT	Inverse SFFT
AWGN	Additive white Gaussian noise

## Appendix A

Using det(I+AB)=det(I+BA), lndet(•) of (23) can be written as:

(A1) $$\begin{array}{cc} A(U_{1}) & =\ln\det\left(GXR_{\alpha }X^{H}G^{H}+R_{w}\right)-\ln\det\left(R_{w}\right)\\ \; & =\ln\det(I+R_{\alpha }^{\frac{1}{2}}X^{H}G^{H}R_{w}^{-1}GXR_{\alpha }^{\frac{1}{2}})\\ \; & =-\ln\det{\left(I+R_{\alpha }^{\frac{1}{2}}X^{H}G^{H}R_{w}^{-1}GXR_{\alpha }^{\frac{1}{2}}\right)}^{-1} \end{array}$$

Using the matrix inversion lemma and the block matrix inversion theorem, the result of Lemma 1 can be obtained.

## Appendix B

Using the matrix inversion lemma, tr(•) of (23) can be written as:

(A2) $$\begin{array}{cc} \; & \mathrm{tr}({\left(GXR_{\alpha }X^{H}G^{H}+R_{w}\right)}^{-1}R_{w})\\ \; & =\mathrm{tr}(\left(R_{w}^{-1}-R_{w}^{-1}GXR_{\alpha }^{\frac{1}{2}}{\left(I+R_{\alpha }^{\frac{1}{2}}X^{H}G^{H}R_{w}^{-1}GXR_{\alpha }^{\frac{1}{2}}\right)}^{-1}R_{\alpha }^{\frac{1}{2}}X^{H}G^{H}R_{w}^{-1}\right)R_{w})\\ \; & =\mathrm{tr}(I-R_{w}^{-1}GXR_{\alpha }^{\frac{1}{2}}{\left(I+R_{\alpha }^{\frac{1}{2}}X^{H}G^{H}R_{w}^{-1}GXR_{\alpha }^{\frac{1}{2}}\right)}^{-1}R_{\alpha }^{\frac{1}{2}}X^{H}G^{H})\\ \; & =NM-\mathrm{tr}(R_{w}^{-1}GXR_{\alpha }^{\frac{1}{2}}{\left(I+R_{\alpha }^{\frac{1}{2}}X^{H}G^{H}R_{w}^{-1}GXR_{\alpha }^{\frac{1}{2}}\right)}^{-1}R_{\alpha }^{\frac{1}{2}}X^{H}G^{H}) \end{array}$$

By applying the matrix inversion lemma again, the second term in the last equality of (A2) can be written as:

(A3) $$\begin{array}{cc} \; & -\mathrm{tr}(R_{w}^{-1}GXR_{\alpha }^{\frac{1}{2}}{\left(I+R_{\alpha }^{\frac{1}{2}}X^{H}G^{H}R_{w}^{-1}GXR_{\alpha }^{\frac{1}{2}}\right)}^{-1}R_{\alpha }^{\frac{1}{2}}X^{H}G^{H})\\ \; & =-\mathrm{tr}(R_{w}^{-1}\left(GXR_{\alpha }X^{H}G^{H}-GXR_{\alpha }X^{H}G^{H}{\left(R_{w}+GXR_{\alpha }X^{H}G^{H}\right)}^{-1}GXR_{\alpha }X^{H}G^{H}\right)) \end{array}$$

By applying the block matrix inversion theorem to (A3), the conclusion of Lemma 2 can be obtained.
